# Requirements for the precision and reproducibility in the efficacy testing of chemical disinfection procedures

**DOI:** 10.3205/dgkh000642

**Published:** 2026-03-20

**Authors:** Kira-Marie Roesch, Marvin Rausch, Felix Droop, Martin Exner, Carola Ilschner, Axel Kramer, Thomas Selhorst, Miranda Suchomel, Nico T. Mutters, Jürgen Gebel

**Affiliations:** 1 University Bonn, University Hostital Bonn, Institut for Hygiene and Public Health, Bonn, Germany; 2 Association for Applied Hygiene (VAH), Bonn, Germany; 3 University of Bonn, Bonn, Germany; 4 Institute of Hygiene and Environmental Medicine, University Medicine Greifswald, Greifswald, Germany; 5 Federal Institute for Risk Assessment, Berlin, Germany; 6 Institute for Hygiene and Applied Immunology, Medical University of Vienna, Vienna, Austria

**Keywords:** disinfectant efficacy testing, method validation, reproducibility, interlaboratory variability, intralaboratory variability, importance ring trials

## Abstract

**Background:**

Assessing the efficacy of chemical disinfectants requires precise and reproducible test procedures. In Germany, the Commission for Infection Prevention and Hygiene in Healthcare and Nursing (KRINKO) at the Robert Koch Institute, the federal government public health institute, recommends that two test reports from independent accredited laboratories, accompanied by expert opinions, to assess the efficacy of disinfectants for use in settings which harbor a special risk for acquiring infections. This requirement, also endorsed by the Association of Applied Hygiene (VAH) for the certification of disinfectants, is based on the inherent statistical variance of microbiological testing methods.

**Methods:**

This paper presents the analysis of data from four VAH-organized ring trials to underline the statistical relevance of the two-test stipulation. Specifically, two quantitative suspension tests were evaluated, one testing efficacy of a test product against *Staphylococcus aureus* according to EN 13727 and one testing efficacy against *Candida albicans* according to EN 13624. In addition, two phase 2 step 2 tests to evaluate the bactericidal efficacy of test products against *Enterococcus hirae *using the quantitative test on non-porous surfaces without mechanical action according to EN 17387 and mycobactericidal efficacy against *Mycobacterium terrae* using the quantitative carrier test for instruments according to EN 14563 were analyzed. A one-way analysis of variance (ANOVA) was performed to assess interlaboratory variations and a Monte-Carlo simulation study was conducted to evaluate the impact of including results from a second laboratory on the reliability of results.

**Results:**

The ANOVA revealed a significant interlaboratory variation. In addition, descriptive analysis indicated substantial intralaboratory variability across trials. The simulation analysis indicates that requiring matching results from two laboratories provides a more conservative and consistent assessment of efficacy. The most marked effects occurred at intermediate concentration-time ratios, where the implementation of the two-laboratory stipulation resulted in a 69–89% reduction in the number of efficacy classifications, thereby significantly mitigating the risk of overly optimistic conclusions. Within the clearly effective or ineffective ranges, however, the differences between the two approaches (one-laboratory rule vs. two-laboratory rule) were minimal.

**Conclusion:**

The study demonstrates considerable interlaboratory variability and illustrates that including results from a second independent laboratory can reduce inconsistencies in efficacy assessments. These findings support the rationale behind requiring two independent test reports. The analysis underlines the importance of ring trials as a key element of quality assurance and method validation in disinfectant efficacy testing.

## Zusammenfassung

**Hintergrund:**

Die Bewertung der Wirksamkeit chemischer Desinfektionsmittel erfordert präzise und reproduzierbare Testverfahren. In Deutschland empfiehlt die Kommission für Infektionsprävention und -hygiene im Gesundheitswesen und in der Pflege (KRINKO) am Robert Koch-Institut der Bundesregierung, dass zwei Testberichte von unabhängigen akkreditierten Laboratorien, begleitet von Gutachten, erforderlich sind, um die Wirksamkeit von Desinfektionsmitteln für den Einsatz in Umgebungen mit besonderem Infektionsrisiko beurteilen zu können. Diese Anforderung, die auch vom Verbund für Angewandte Hygiene (VAH) für die Zertifizierung von Desinfektionsmitteln unterstützt wird, basiert auf der statistischen Varianz mikrobiologischer Testmethoden.

**Methoden:**

In diesem Artikel werden die Daten aus vier vom VAH organisierten Ringversuchen analysiert, um die statistische Relevanz der geforderten zwei unabhängigen Tests zu unterstreichen. Hierzu wurden zwei quantitative Suspensionstests ausgewertet, einer zur Prüfung der Wirksamkeit eines Testprodukts gegen *Staphylococcus aureus* gemäß EN 13727, der andere zur Prüfung der Wirksamkeit gegen *Candida albicans* gemäß EN 13624. Darüber hinaus wurden zwei praxisnahe Phase-2-Stufe-2-Tests zur Bewertung der bakteriziden Wirksamkeit von Testprodukten gegen *Enterococcus hirae* unter Verwendung des quantitativen Tests auf nicht porösen Oberflächen ohne mechanische Einwirkung nach EN 17387 und der mykobakteriziden Wirksamkeit gegen *Mycobacterium terrae* unter Verwendung des quantitativen Keimträgertests für Instrumente gemäß EN 14563 analysiert. Zur Bewertung der Laborunterschiede wurde eine einseitige Varianzanalyse (ANOVA) durchgeführt. Zur Bewertung der Auswirkung der Einbeziehung der Ergebnisse eines zweiten Labors auf die Zuverlässigkeit der Ergebnisse wurde eine Monte-Carlo-Simulationsstudie durchgeführt.

**Ergebnisse:**

Die ANOVA ergab eine signifikante Variation zwischen den Laboren. Darüber hinaus zeigte die deskriptive Analyse eine erhebliche Variabilität der Ergebnisse innerhalb der Labore. Die Simulationsanalyse ergab, dass die Forderung nach übereinstimmenden Ergebnissen aus zwei Laboren eine konservativere und konsistentere Bewertung der Wirksamkeit ermöglicht. Die deutlichsten Auswirkungen traten bei einem intermediären Konzentrations-Zeit-Verhältnis auf, wo die Anwendung der Zwei-Labor-Regel zu einer Verringerung der Anzahl der Wirksamkeitsklassifizierungen um 69–89% führte und damit das Risiko übermäßig optimistischer Schlussfolgerungen erheblich verringerte. Innerhalb der eindeutig wirksamen oder unwirksamen Bereiche waren die Unterschiede zwischen den beiden Ansätzen (Ein-Labor-Prüfung vs. Zwei-Labor-Prüfung) jedoch minimal.

**Schlussfolgerung:**

Die Studie zeigt eine erhebliche Variabilität zwischen den Laboren und verdeutlicht, dass die Einbeziehung der Ergebnisse eines zweiten unabhängigen Labors Unstimmigkeiten bei der Wirksamkeitsbewertung reduzieren kann. Diese Ergebnisse stützen die Begründung für die Forderung nach zwei unabhängigen Testberichten. Die Analyse unterstreicht die Bedeutung von Ringversuchen als Schlüsselelement der Qualitätssicherung und Methodenvalidierung bei der Prüfung der Wirksamkeit von Desinfektionsmitteln.

## Introduction

When assessing the efficacy of a disinfection procedure for awarding a certificate of efficacy, the Association for Applied Hygiene (VAH) requires the submission of two test reports from two independent accredited test laboratories [1]. This approach addresses the inherent statistical variation in microbiological disinfectant tests, with the aim of ruling out incorrect conclusions regarding the efficacy of a product when used in the claimed concentration-time ratio(s). To precisely determine the extent of statistical deviations, VAH ring trials have been carried out for more than 15 years, involving numerous experienced laboratories. The ring trials evaluate technical competence of the laboratory carried out under standardized specifications to ensure quality control and accreditation compliance. The detailed test reports are published on the VAH website [2]. 

In Europe, the Technical Committee for Chemical Disinfectants and Antiseptics (CEN TC 216) implemented a two-phase approach for disinfection testing. The first phase consists of quantitative suspension tests, which assess the basic activity of a disinfectant. The second phase involves quantitative suspension tests under conditions representative of practical use (step 1) and tests which evaluate efficacy under simulated practice conditions (step 2) such as carrier tests for hard surfaces. This two-phase approach ensures that disinfectants meet both theoretical and practical performance criteria. CEN TC 216 has commissioned ring trials for mandated standards such as EN 13727 [3], EN 13624 [4] and EN 14348 [5] to validate test methods and establish precision parameters. To further enhance proficiency testing, a Ring Trial Task Force was established in 2022, operating across CEN Working Groups – the Horizontal Working Group (WG5) and three Working Groups responsible for the Medical (WG1), Veterinary (WG2) and Food Hygiene, Domestic and Institutional (WG3) market sectors. This task force has a crucial role for using data from ring trials by defining a clear methodological framework for ring trials, ensuring the publication of results, and developing proposals for the revision of existing efficacy test standards. The following section summarizes the information on the required precision of results as recommended in the pertaining European standards. 

EN 14885 [6], the standard concerning the application and interpretation of standards elaborated by CEN TC 216, specifies that test methods must be verified through ring trials. It further requires that the precision or the respective pass limits enables a product evaluation without the need to assess of the inaccuracy of the respective test. Recommendations to improve precision of test results comprise maintaining quality assurance systems, regular participation in ring trials, test repetitions, inclusion of internal standards and performing tests in multiple laboratories. 

The calculations of the two statistical evaluations included in the EN standards (Annex E of EN 13727 [3] and EN 13624 [4]) show that the test must be repeated 4 to 6 times in a test laboratory to generate a precision result with a standard deviation of ±1 lg. No information on the requirements for result precision can be found in EN 14348 [5], EN 14561 [7], EN 14562 [8] and EN 14563 [9]. 

The VAH requirements for product certification stipulate that applicants must submit test results from both phases 1 and phase 2 tests, covering all activity spectra where applicable. These results must be provided by two independent, accredited laboratories, phase 2 step 2 tests being performed in duplicate. In addition, laboratories conducting efficacy tests for the VAH certification process are obligated to regularly participate in ring trials to ensure reliability and comparability of results. These conditions are aligned with recommendations of EN 14885 [6], which outlines the application of European standards for chemical disinfectants and antiseptics, and highlight the necessity of laboratory accreditation (e.g., DIN EN ISO/IEC 17025) [10]. Accreditation serves as a critical safeguard for the independence, reproducibility, and quality assurance of the testing process. 

The European Chemicals Agency (ECHA) employs a different approach for biocide evaluation with regard to its requirements for test laboratories and repetitions (Guidance on the BPR: Volume II Parts B+C – Version 6.0 August 2023) [11]. Typically, the evaluation procedure by ECHA relies on test reports from a single laboratory and does not require laboratory accreditation (only a quality management system certificate). There is also no requirement for the testing laboratory to be independent of the disinfectant manufacturer. Theoretically, the applicant can therefore test its own product and submit these results for the assessment procedure. Repetition of the test results in the respective test laboratory is referred to under 5.4.0.4.3 Data requirements – repetitions but is not explicitly required [10]. 

## Method

### Data set

Four ring trials, both for phase 2 step 1 and phase 2 step 2 tests, were selected based on their bactericidal, yeasticidal and mycobactericidal activity [12], [13], [14], [15].

All test products were provided from a single batch for all participants by the VAH. The aim of the tests was to determine the reduction for each test product at different product concentrations and contact times under the specified test conditions. The interlaboratory reproducibility of the test protocol and the interlaboratory reproducibility of the determined activity were checked. Based on preliminary tests of the VAH reference laboratory, ineffective concentration-time ratios, concentration-time ratios in the so-called “intermediate effective” range, which is at the efficacy limit, and effective concentration-time ratios were to be found. 

Graphical representations of laboratory results were generated based on the individual reduction values obtained from VAH-organized ring trials. Data from participating laboratories were compiled and sorted by mean lg-reduction values for each test condition. Graphs were created using GraphPad Prism (version 8.0.2, GraphPad Software, San Diego, CA, USA). 

### Assessment of interlaboratory variation

To assess whether the laboratory had a significant influence on the reductions, a one-factor analysis of variance (ANOVA) with the factor “laboratory” was performed (α=0.05). An ANOVA was used to statistically test the equality of means using F-tests. Here the global F-test was used to test for overall differences among laboratory means. The statistical software R version 4.3.3 was used for the analysis.

### Assessment of intralaboratory variation

Variability arises both within individual laboratories and between laboratories, raising the question of whether efficacy assessments based on a single laboratory are sufficiently reliable. Against this background, the present study compares two decision rules:

- One-laboratory rule: Within the Monte-Carlo simulation, this regulatory approach designates a single laboratory – selected at random – as the sole authority for the efficacy assessment.
- Two-laboratory rule: Under this rule, within the Monte-Carlo simulation, a substance is classified as effective only if both independent laboratories unanimously report it as effective.

The proportion of laboratories with a statement effective (lg-R≥5 for bactericidal efficacy and lg-R≥4 for yeasticidal and mycobactericidal efficacy) and not effective (lg-R<5 accordingly lg-R<4) was then determined. All statements from the laboratories were then compared in pairs and it was checked whether two laboratories made the same statement or not. 

Based on the pairwise comparisons, a binomially distributed data set was obtained, which contained a “1” if the laboratories came to an unequal result and a “0” if the result were the same. 

The following simulation was carried out 10,000 times to analyse the change in the statement when a second laboratory was added:

1. Choose a laboratory at random and write down the statement
2. Randomly select a second laboratory and compare the statement with 1.
3. The decision of the laboratory (effective or ineffective) is noted under 1. and under 2. The final decision effective is only noted if both laboratories have decided that the product is effective. Otherwise it is declared ineffective.
4. Insert the decision into the four-field table according to the following pattern (Table 1 (Tab. 1)):

After performing the simulation, a four-field table with n=10,000 entries was obtained. An odds ratio (OR) was then calculated for this four-field table. This provided information on how the odds ratio of obtaining an effective result changed when an additional laboratory was included. The significance of the OR was then tested using a statistical test (α=5%), which usually yielded to a significant result due to the high number of simulations runs.

### Odds and odds ratio

In the context of this paper, odds describe the ratio of the probability that a product is classified as effective to the probability that it is classified as ineffective when the decision is based on the results of one laboratory or two laboratories, respectively. The odds ratio (OR) further describes the relationship between these two odds values and is used here as a descriptive measure to compare the relative strictness of the two decision rules. If the odds ratio is greater than 1, the odds of declaring the product effective are higher when considering the results of two laboratories; if it is lower than 1, they are higher when considering the results of one laboratory. Odds and odds ratio are calculated using a four-field table (see section „Monte-Carlo simulation“ above). The odds for two labs are calculated using A/C, and for one lab using B/D. The odds ratio is then determined using the following formula: 

(A/C)/(B/D)=(A*D)/(B*C)

In the present study OR is used to quantify the relative difference in the odds of an efficacy classification between two decision rules (one-laboratory versus two-laboratory assessment).

## Results

### Distribution of laboratory results in ring trials

An overview of the lg-reductions of the intermediate effective range of each ring trial is compiled in Table 2 (Tab. 2). All ring trials, show a great repeatability within the laboratories with repeatability SD<0.5, which is a common value set for the internal lab deviation value in disinfection area. In contrast, the reproducibility between laboratories is >1.0 for three out of four ring trials, which should be better. 

**Figure 1:**
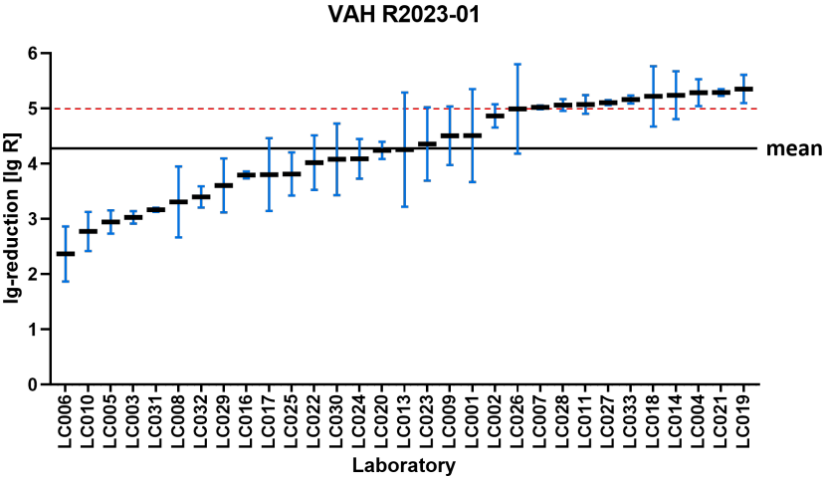
VAH R2023-01 – reduction of *Staphylococcus aureus* according to EN 13727 (glutaraldehyde; 0.05% – 5 min) sorted by laboratory mean values of lg reduction values (the horizontal red dashed line indicates the required bactericidal efficacy threshold of ≥5 lg-reduction; the overall mean reduction of all laboratory results is marked by the black line=4.27±0.32 lg)

**Figure 2:**
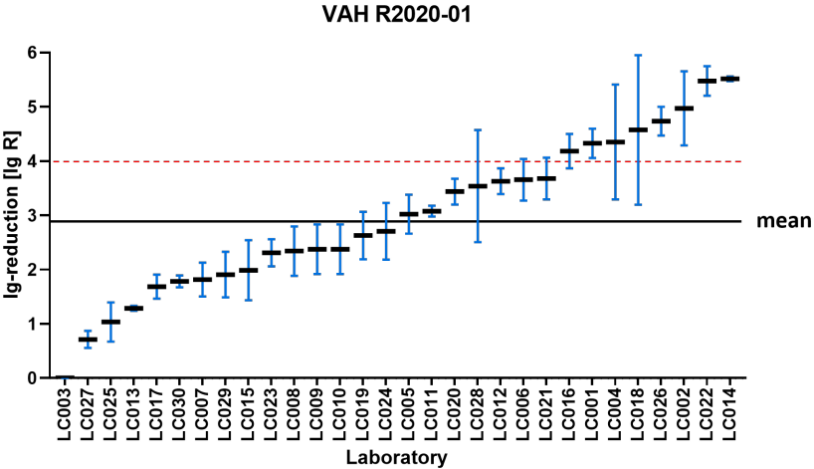
VAH R2020-01 – reduction of *Candida albicans* according to EN 13624 (product A; 0.2% – 15 min) sorted by laboratory mean lg reduction values (the horizontal red dashed line indicates the required yeasticidal efficacy threshold of ≥4 lg-reduction; the overall mean of all laboratory results is marked by the black line=2.89±0.46 lg)

**Figure 3:**
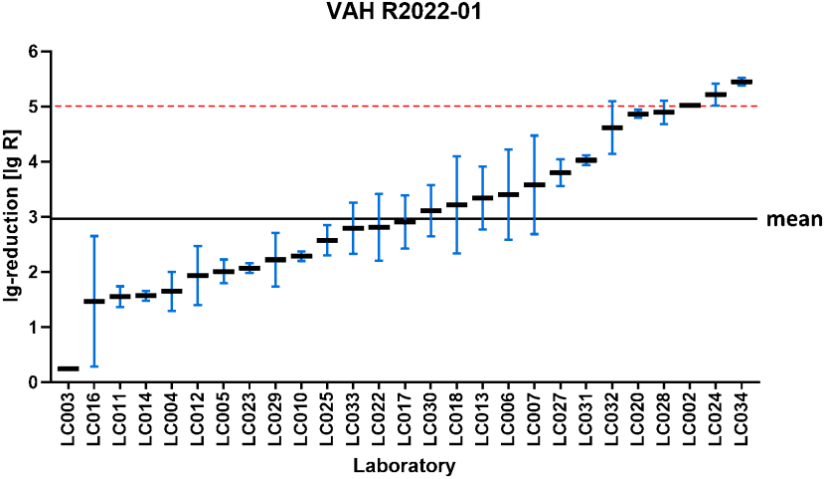
VAH R2022-01 – reduction of *Enterococcus hirae* according to DIN EN 17387 (product A: 40% – 5 min) sorted by laboratory mean lg reduction values (the horizontal red dashed line indicates the required bactericidal efficacy threshold of ≥5 lg-reduction; the overall mean lg reduction of all laboratory results is marked by the black line=2.98±0.58 lg)

**Figure 4:**
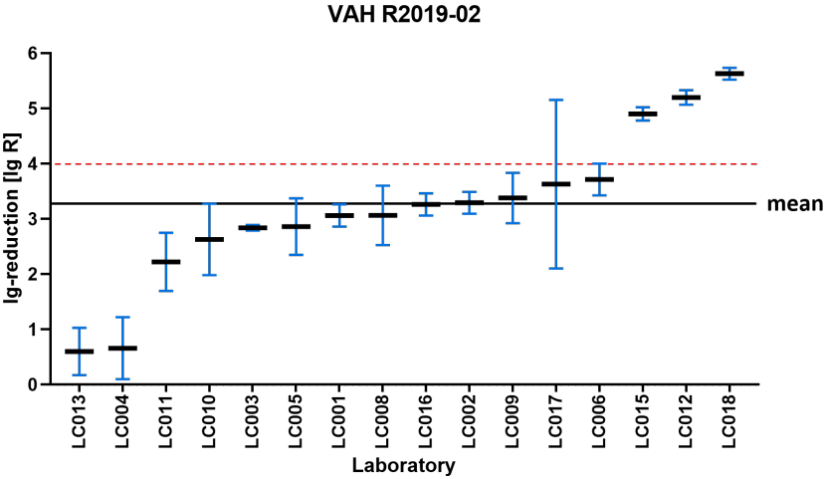
VAH 2019-02 – reduction of *Mycobacterium terrae* according EN 14563 (product A: 1%–30 min) sorted by laboratory mean lg values (the horizontal red dashed line indicates the required mycobactericidal efficacy threshold of ≥5 lg-reduction; the overall mean lg reduction values of all laboratory results is marked by the black line=3.34±0.66 lg)

Figure 1 (Fig. 1), Figure 2 (Fig. 2), Figure 3 (Fig. 3), and Figure 4 (Fig. 4) display the distribution of reduction values reported by the participating laboratories for the respective intermediate concentration-time ratio of each ring trial. The data show considerable interlaboratory variability, with differences visible across the intermediate effective concentration-time ratio. These graphical representations illustrate the spread of mean log_10_ reduction values per laboratory and highlight the variability that underpins the subsequent statistical analyses. 

### ANOVA

The data underlying the ANOVA is based on the above listed VAH ring trials. For each ring trial the ineffective, the intermediate effective and the effective range with each number of laboratories classifying the product as effective is listed in the table below (see Table 3 (Tab. 3)). With this data, the ANOVA using F-tests analysis was performed. The F-statistic is the ratio of the variance between sample means to the variance within samples. The results are summarized in Table 4 (Tab. 4). The values in the F-test are the degrees of freedom and are used as the numerator and denominator to calculate the F-value, which is then used to calculate the p-value.

The analysis of the reductions at different concentration-time ratios using ANOVA (see Table 4 (Tab. 4)) shows that the influence of the laboratory on the reductions in all three efficacy ranges (ineffective, intermediate effective and effective) can be described as significant (p-values all<0.001) for all VAH ring trials.

### Monte-Carlo simulation of the ineffective range 

For the ineffective range of each ring trial, the proportion of laboratories in VAH ring trials with a decision of ineffective is 100%. No simulation was carried out here, as the decision ineffective was unanimous (see Table 3 (Tab. 3)). 

### Monte-Carlo simulation of intermediate effective and effective range

For the intermediate effective and the effective range of each ring trial, the proportion of laboratories in VAH ring trials with a decision of effective is shown in Table 3 (Tab. 3). On this basis, the pairwise comparisons that yielded different results, were calculated. In other words, all those comparisons in which one laboratory stated effective and the other said ineffective, or vice versa. The percentage of pairwise comparisons that produced divergent results is presented in Table 5 (Tab. 5) for the intermediate effective and the effective ranges. The following four-field Table 6 (Tab. 6) and Table 7 (Tab. 7) show results of the intermediate effective and the effective range from the Monte-Carlo simulation. The results are described below. 

### R2023-01

If only the decision of one laboratory is taken into account for the intermediate effective range, the product is declared effective in 3,232/10,000 (32.3%) of cases. If a second laboratory is used, this probability is only 1,026/10,000 (10.3%), referring to the proportion of decisions that have been declared effective (see Table 6 (Tab. 6)).

The resulting odds ratio (OR) is 0.24. This means that the odds of the product being declared effective are reduced by 76% (1–0.24=0.76) if the decision of a second laboratory is used in the assessment, referring to the OR. 

In the effective range, the decision effective is made by one laboratory in 8,583/10,000 (85.8%) of cases. The addition of a second laboratory reduces this proportion to 7,277/10,000 (72.7%), referring to the proportion of decisions that have been declared effective (see Table 7 (Tab. 7)). 

This corresponds to an OR of 0.44, indicating lower odds of a product being classified as effective in the two-laboratory setting compared to the one-laboratory setting.

### R2020-01

If only the decision of one laboratory is considered for the intermediate effective range, the product is declared effective in 2,548/10,000 (25.5%) of cases. If a second laboratory is used, this probability is only 611/10,000 (6.1%), referring to the proportion of decisions that have been declared effective (see Table 6 (Tab. 6)). 

The resulting OR is 0.19. This means that the odds of the product being declared effective is reduced by 81% if the decision of a second laboratory is used in the assessment, compared to the one-laboratory setting. 

In the effective range, the decision effective is made by one laboratory in 6,465/10,000 (64.5%) of cases. The addition of a second laboratory reduces this proportion to 4,101/10,000 (41.0%), referring to the proportion of decisions that have been declared effective (see Table 7 (Tab. 7)). 

This corresponds to an OR of 0.38, indicating lower odds of a product being classified as effective in the two-laboratory setting compared to the one-laboratory setting.

### R2022-01

If only the decision of one laboratory is considered for the intermediate effective range, the product is declared effective in 1,068/10,000 (10.7%) of cases. If a second laboratory is used, this probability is only 82/10,000 (0.8%), referring to the proportion of decisions that have been declared effective (see Table 6 (Tab. 6)). 

The resulting OR is 0.07. This means that the odds of the product being declared effective is reduced by 94% if the decision of a second laboratory is used in the assessment. 

In the effective range, the decision effective is made by one laboratory in 8,915/10,000 (89.2%) of cases. The addition of a second laboratory reduces this proportion to 7,895/10,000 (79.0%), referring to the proportion of decisions that have been declared effective (see Table 7 (Tab. 7)). 

This corresponds to an OR of 0.46, indicating lower odds of a product being classified as effective in the two-laboratory setting compared to the one-laboratory setting.

### R2019-02

If only the decision of one laboratory is considered for the intermediate effective range, the product is declared effective in 1,865/10,000 (18.7%) of cases. If a second laboratory is used, this probability is only 250/10,000 (2.5%), referring to the proportion of decisions that have been declared effective (see Table 6 (Tab. 6)). 

The resulting OR is 0.11. This means that the odds of the product being declared effective is reduced by 89% if the decision of a second laboratory is used in the assessment. 

In the effective range, the decision effective is made by one laboratory in 7,563/10,000 (75.6%) of cases. The addition of a second laboratory reduces this proportion to 5,590/10,000 (55.9%), referring to the proportion of decisions that have been declared effective (see Table 7 (Tab. 7)). 

This corresponds to an OR of 0.41, indicating lower odds of a product being classified as effective in the two-laboratory setting compared to the one-laboratory setting.

### Graphical visualization

**Figure 5:**
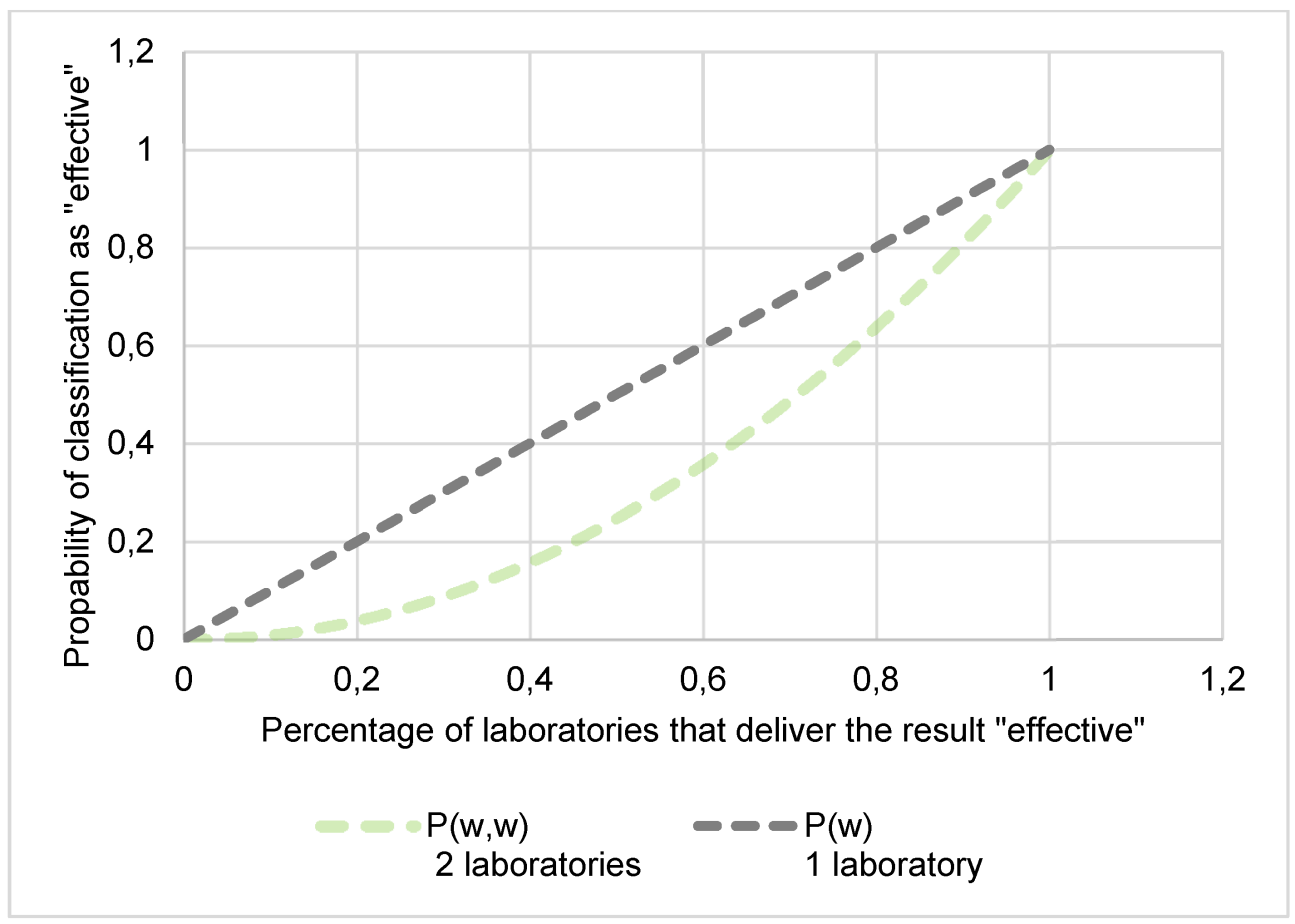
Test strategy one-laboratory rule compared to test strategy two-laboratory rule

The graphical presentation visualized the test strategy of one-laboratory rule compared to the two-laboratory rule. This approach can be used to show under which circumstances one- laboratory and the two-laboratory rule differ greatly and in which areas they converge more closely. On this basis, it becomes clear that the one-laboratory rule and the two-laboratory rule do not differ in terms of the reliable efficacy of a disinfectant. In the uncertain intermediate range, however, the two-laboratory rule proves to be more conservative (see Figure 5 (Fig. 5)).

## Discussion

The European Committee for Standardization (CEN) has established a comprehensive framework for evaluating disinfectant testing, incorporating standardized test methods for various applications. This framework mandates the use of surrogate test organisms representing different microbial classifications, specifies microbicidal and virucidal efficacy claims and takes into account practical application conditions. These stringent requirements ensure that disinfectant efficacy claims are supported by robust scientific evidence and remain relevant to real-world disinfection practices. 

The assessment of disinfectant efficacy requires a comprehensive approach combining suspension tests and tests under simulated practical conditions (phase 2, step 2 tests). While suspension tests provide valuable initial data, phase 2, step 2 tests more accurately simulate real-world use conditions by accounting for surface types and materials, organic soil loads, environmental conditions (e.g. temperature and humidity), and application methods [6]. Their superior predictive value and reproducibility make them indispensable for verifying disinfectant efficacy.

The significant interlaboratory variability remains a major challenge in microbiological efficacy testing. Differences in test organism preparation, media and reagent quality, technician experience and technique, as well as differences in equipment and environmental conditions contribute to inconsistent results, particularly in the intermediate efficacy range [16]. Recent interlaboratory ring trials, such as published by Suchomel et al., demonstrate substantial variability despite adherence to EN 1500, confirming the need for enhanced standardization and reproducibility measures [17].

Statistical analyses in the present study, including ANOVA and simulation models, revealed significant heterogeneity in reduction rates across the laboratories of all four VAH ring trials included in this publication. Confidence intervals confirmed that this variability, particularly within the borderline range between efficacy and inefficacy, directly impacts the reliability of the product evaluations. The simulation study provides compelling evidence for the value of including a second laboratory’s results in disinfectant efficacy assessments. Multi-laboratory testing improved the robustness of efficacy assessments by reducing the influence of interlaboratory variability, thereby decreasing the likelihood of overly optimistic efficacy classifications under standardized conditions. In statistical terms, this denotes increased robustness of efficacy classifications against operator-, instrument-, and environment-related variability. In ring trials, this leads to more consistent and conservative assessment outcomes. With improved accuracy of efficacy, the likelihood of false-positive results, which occurs when a product is classified as “effective” despite failing to achieve the required microbial reduction threshold, is reduced. Greater consistency of classification outcomes across laboratories indicates a lower probability of false-positive classification.

High robustness of efficacy assessments ensures that the odds ratio primarily reflects differences in classification outcomes between decision rules rather than noise or interlaboratory bias, thereby strengthening the interpretability of the results. Conversely, high rates of false-positive classifications may distort the odds ratio by inflating the odds of efficacy classification under less stringent decision rules.

In the first ring trial (R2023-01), a second bacterial suspension test increased the robustness of efficacy classification, corresponding to a 76% reduction in the odds of classifying products as effective within the intermediate effective range. Similarly, the second suspension test (R2022-01), conducted with yeasts, and resulted in an 81% reduction in the odds of efficacy classification in this range. The impact was even more pronounced in simulated practical tests. The quantitative test on non-porous surfaces without mechanical action against *E. hirae* (R2022-01) showed a 94% reduction, while the quantitative carrier test for instrument disinfection (by immersion) against *M. terrae* (R2019-02) demonstrated 89% reduction in the odds of efficacy classification within the intermediate effective range. These results underscore the critical role of independent evaluation in ensuring the robustness of efficacy classification. Most importantly, our data indicate that the risk of misinterpretation is particularly pronounced in phase 2, step 2 tests, where practical conditions introduce additional sources of variability. The substantial improvement in classification odds when a second laboratory was included highlights the necessity of dual-laboratory validation in all efficacy levels. 

Notably, the benefit of a second laboratory assessment extends beyond borderline efficacy cases. Even in clearly effective ranges, the robustness of efficacy classification increased substantially, corresponding to a 62% reduction in the odds of classifying products as effective under a single-laboratory decision rule. The results of this study support the recommendations of EN 14885 [6] and VAH guidelines [1], which emphasize quality assurance through regular participation in ring trials and the use of accredited laboratories. Such measures are crucial in view of the regulatory requirement to employ low active-ingredient concentrations to minimize chemical exposure under the European Union (EU) Biocidal Products Regulation (EU No. 528/2012) [18]. As many disinfectants operate within the critical threshold between efficacy and inefficacy – referred to as the intermediate range in this study –, precise and reliable evaluation methods are essential. 

Comparable approaches are applied internationally. The requirements of the German Veterinary Medical Association (DVG) [19], which issues certificates for biocides used in the veterinary sector and food industry, and the Austrian Society for Hygiene, Microbiology, and Preventive Medicine (ÖGHMP) [20], closely mirror those of the VAH. Applicants must provide a test report and an expert evaluation of the results, particularly with respect to the concentration–time relation to be certified and listed. In addition, a confirmatory benchmark test must be conducted by an independent second laboratory to validate the findings [1], [19]. Similarly, the OECD method for surface disinfection mandates three independent test runs in one to three different test laboratories [21], while regulatory bodies such as the United States Environmental Protection Agency (EPA) and the U.S. Food and Drug Administration (FDA) require three independent tests on separate product batches [22]. In the EPA handbook, 16 of the 42 antimicrobial testing methods require three independent tests on separate product batches (the source is cited for only one method as an example [22]). These requirements reflect the general tendency to emphasize the need for a solid and reproducible database for assessing the efficacy of disinfectants to ensure public health and safety.

Conversely, the European Chemicals Agency (ECHA) currently accepts a single test run for efficacy confirmation [11], an approach increasingly questioned given the substantial interlaboratory variability demonstrated in recent studies. In the assessment of the KRINKO on requirements for disinfectants for use in infection-sensitive areas, the following requirements, which go beyond the biocidal product authorization, must be met for the use of disinfectants in areas sensitive to infection hygiene [23]:

- The reproducibility of the results must be confirmed by two independent laboratories.
- Requirements for the replication of efficacy tests must be met.

Our results provide evidence supporting KRINKO’s dual-test requirement and the VAH’s two-laboratory validation policy, while challenging the adequacy of single-laboratory verification. Similar conclusions were drawn in the study on the development and validation of the single tube method (STM) for efficacy testing of antimicrobial products against biofilms, which shows good overall reproducibility, but wide variations in results between different laboratories, especially when evaluating the lg-reductions in viable bacteria by different antimicrobials [24]. 

Ensuring reliable efficacy data is essential for balancing the need for minimal biocide concentrations with the requirement for effective infection prevention. Interdisciplinary collaboration between academia, industry and regulatory authorities remains critical to ensure that efficacy testing evolves with emerging pathogens and novel disinfectant technologies. 

## Conclusion

This study demonstrates, based on VAH ring trial data, that the inclusion of a second independent laboratory significantly improves the precision and reproducibility of disinfectant efficacy assessments. This effect was observed consistently across suspension tests and tests simulating practice conditions, various microorganisms, and soiling conditions, highlighting its general applicability. The statistical analyses, particularly ANOVA and Monte-Carlo simulation, provide robust evidence that single-laboratory testing is insufficient in capturing the inherent variability of microbiological efficacy tests.

The findings support the requirements of VAH and KRINKO for dual-laboratory validation and are consistent with similar approaches adopted by DVG, OECD, ÖGHMP, EPA, and FDA. While ECHA currently accepts a single-laboratory testing approach [11], the results of the present analyses do not support this recommendation, as they demonstrate that single-laboratory assessments systematically underestimate interlaboratory variability. In essence, requiring independent confirmation across multiple laboratories is not merely a statistical refinement but a prerequisite for reliable disinfectant approval, robust infection control, and sustained public health protection. In this context it is also important to note that sub-lethal disinfectant exposure may promote microbial tolerances or resistance [25], highlighting the necessity for continued surveillance, adaptive regulation, and innovation in disinfectant development. This is also reflected in the latest Guidance Document, *Guidance on the evaluation of resistance to biocidal active substances and products*, where ECHA proposes that resistance management strategies should include avoiding the application of biocides in sub-lethal doses [26].

Consequently, the approval of disinfectants to be placed on the market and their ongoing re-evaluation/post-market surveillance requires a multidisciplinary approach that includes microbiological efficacy testing, and ecotoxicological assessments. The implementation of standards that require the inclusion of data from multiple independent sources is key to ensuring the reliability and efficacy of disinfection procedures in real clinical practice.

## Notes

### Authors’ ORCIDs:

- Roesch K: https://orcid.org/0009-0000-3494-9191
- Rausch M: https://orcid.org/0000-0003-1562-4337
- Exner M: https://orcid.org/0000-0002-6383-7866
- Ilschner C: https://orcid.org/0009-0006-4083-7405
- Kramer A: https://orcid.org/0000-0003-4193-2149
- Selhorst T: https://orcid.org/0000-0003-0969-1533
- Suchomel M: https://orcid.org/0000-0001-8758-9652
- Mutters NT: https://orcid.org/0000-0002-0156-9595
- Gebel J: https://orcid.org/0000-0001-9328-3174

### Funding 

This work was funded by the Association for Applied Hygiene (VAH), Germany. 

### Acknowledgments 

The authors are grateful for the input provided by all laboratories which participated in the VAH ring trials.

### Authors’ contributions

The authors Roesch and Rausch contributed equally.

### Competing interests 

The authors declare that they have no competing interests.

## Figures and Tables

**Table 1:**
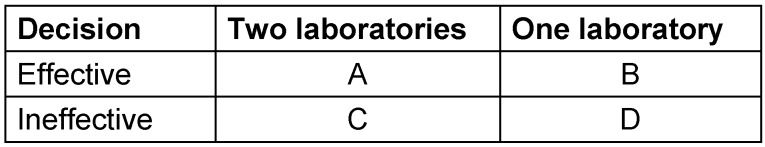
Construction of a four-field panel for simulation

**Table 2:**
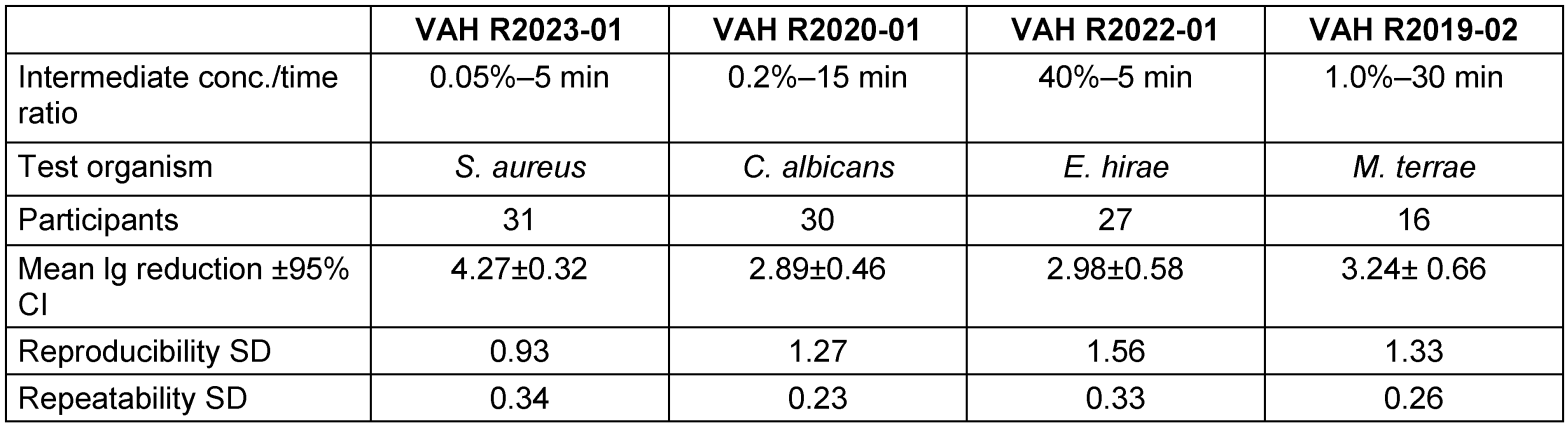
Statistical parameters for the reduction of test organisms with test product according to standards (repeatability SD: standard deviation of repeatability; reproducibility SD: standard deviation of reproducibility, CI: confidence interval)

**Table 3:**
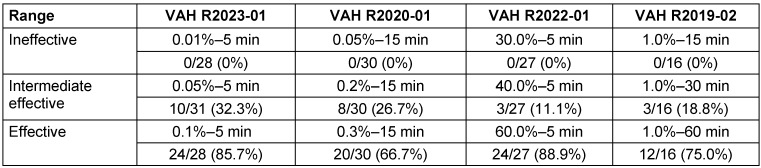
Number of laboratories in the different ring trials and ranges which classified the product as effective

**Table 4:**
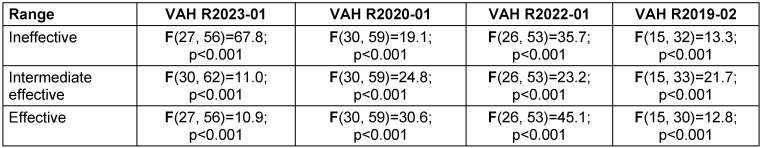
Results from the ANOVA evaluation using F-tests of each VAH ring trial and each ineffective, intermediate effective and effective range with the F-test and the p-value

**Table 5:**
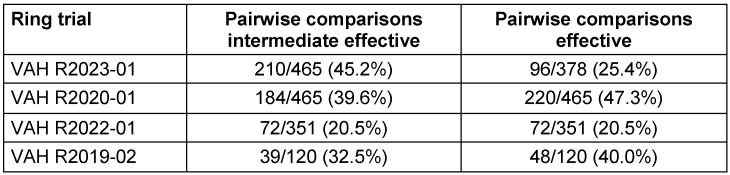
All comparisons in which one laboratory said effective and the other ineffective or vice versa for the intermediate effective and the effective range of each ring trial

**Table 6:**
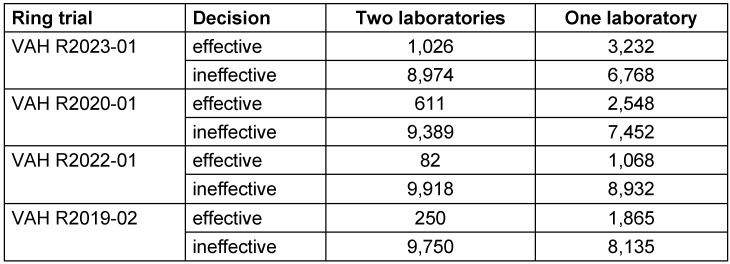
Four-field table of the simulation for the intermediate effective range

**Table 7:**
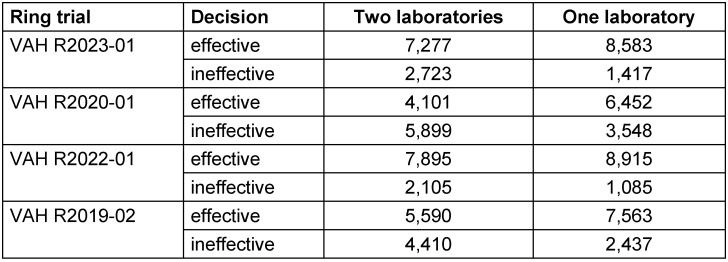
Four-field table of the simulation for the effective area
